# The Pre- and Postoperative FIB-4 Indexes Are Good Predictors to the Outcomes of HBV-Related HCC Patients after Resection

**DOI:** 10.1155/2019/8945798

**Published:** 2019-12-01

**Authors:** Meng-Yun Tsai, Yi-Hao Yen, Pao-Yuan Huang, Fai-Meng Sou, Chih-Che Lin, Wei-Ru Cho, Hsin-Ming Wang, Ding-Wei Chen, Kuo-Chin Chang, Cheng-Kun Wu, Tsung-Hui Hu, Ming-Chao Tsai

**Affiliations:** ^1^Department of Pulmonary and Critical Care Medicine, Chang Gung Memorial Hospital, Kaohsiung, Taiwan; ^2^Division of Hepato-Gastroenterology, Department of Internal Medicine, Chang Gung Memorial Hospital, Kaohsiung, Taiwan; ^3^Liver Transplantation Center and Department of Surgery, Kaohsiung Chang Gung Memorial Hospital and Chang Gung University College of Medicine, Kaohsiung, Taiwan; ^4^Center for Translational Research in Biomedical Sciences, Liver Transplantation Program and Department of Surgery, Kaohsiung Chang Gung Memorial Hospital and Chang Gung University College of Medicine, Kaohsiung, Taiwan; ^5^Graduate Institute of Clinical Medical Sciences, Chang Gung University College of Medicine, Taiwan

## Abstract

**Background and Aim:**

Liver fibrosis is associated with the prognosis of patients with hepatocellular carcinoma (HCC) after resection. The fibrosis-4 (FIB-4) index is an accurate and noninvasive marker to determine the degree of liver fibrosis. Here, we evaluated the effect of pre- and postoperative FIB-4 index in predicting the outcomes after resection of HCC in patients who have chronic hepatitis B (CHB) infection.

**Methods:**

A total of 534 CHB patients with HCC who received curative hepatectomy between 2001 and 2016 at Kaohsiung Chang Gung Memorial Hospital, Taiwan, were enrolled in this study. The impact of the FIB-4 index (preoperative and the 1^st^ year after operation) on overall survival (OS) and recurrence-free survival (RFS) was evaluated.

**Results:**

There was a significant association between the preoperative FIB-4 index and Metavir fibrosis stage (*p* < 0.01). The multivariate analysis showed that preoperative FIB‐4 > 2 is an independent risk factor for RFS and OS after HCC curative resection [hazard ratio (HR), 1.902; 95% CI, 1.491–2.460; *p* < 0.001, and HR, 2.207; 95% CI, 1.420–3.429; *p* < 0.001, respectively]. Notably, preoperative FIB-4 is also an independent risk factor for RFS (HR, 1.219; *p* = 0.035) in noncirrhotic patients. Furthermore, patients had deteriorated FIB-4 1 year after operation [definition: the value (the 1^st^ year FIB‐4 after operation minus preoperative FIB‐4) > 1] and had an adverse outcome in RFS and OS (*p* < 0.001, both).

**Conclusion:**

The pre and postoperative FIB-4 indexes are useful clinical markers to predict the prognosis in HBV-HCC patients after curative hepatectomy.

## 1. Introduction

Hepatocellular carcinoma (HCC), the fifth most common cancer worldwide, is a major health problem which can result from chronic inflammation induced by viral infection, such as hepatitis B and C, high intake of alcohol, and metabolic syndrome [1, 2]. The incidence of HCC has increased over the past decade, and it is characterized by a high frequency of fibrosis and cirrhosis, which may impact the host inflammatory microenvironment [2]. Surgical resection remains the most effective treatment for patients with early stage HCC and who are with well-preserved liver function. However, even after complete HCC resection, the carcinogenic tissue microenvironment in the remnant liver can give rise to recurrent HCC. Thus, early detection and prevention of HCC recurrence are the most impactful strategies to improve HCC patients after complete resection.

HCC patients with advanced fibrosis or cirrhosis have a poor prognosis; thus, the preoperative assessment of liver fibrosis and cirrhosis is crucial for optimizing patient prognosis [3]. Currently, the gold standard for diagnosing and staging hepatic fibrosis is liver biopsy, but in fact, biopsy is impractical because of its invasiveness and complications. The accuracy of liver biopsy is also severely compromised by intra- and interobserver variation as well as sampling error [4]. Numerous serologic tests have been developed to detect liver fibrosis. One of them is the fibrosis-4 index (FIB-4) scoring which is the most widely used serum marker [5, 6]. This model has already been used to evaluate liver fibrosis in patients who are chronically infected with HBV or HCV, even in HCC patients scheduled to undergo liver resection [7–9]. However, the prognostic role of the pre- and postoperative FIB-4 score and the variation of FIB-4 score in HBV-HCC patients after resection was likely underestimated.

In the present study, we evaluate the effect of postoperative FIB-4 changes in predicting the outcomes in patients with HBV-related HCC with BCLC stage 0 or A after curative resection.

## 2. Patients and Methods

### 2.1. Patients and Follow-Up

**Figure 1:**
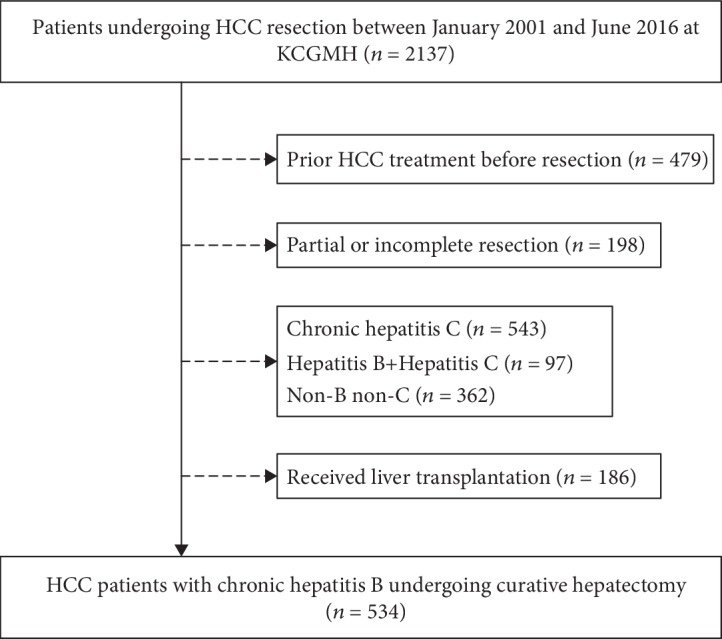
Schematic flowchart of the enrollment process.

This is a retrospective study conducted at Kaohsiung Chung Gung Memorial Hospital, Taiwan. This study complies with the standard of the Declaration of Helsinki and current ethical guidelines, and this study has been approved by the Ethic Committee of Chang Gung Memorial Hospital. Written informed consents were obtained from all patients. Between January 2001 and June 2016, a total of 2137 patients who had HCC and received resection were enrolled at first. Patients had prior HCC treatment before resection (*n* = 479), partial or incomplete resection (*n* = 918), hepatitis C (*n* = 543), hepatitis B plus hepatitis C (*n* = 97), and patients without hepatitis B nor hepatitis C (*n* = 362), and those who underwent liver transplantation (*n* = 186) were excluded. Finally, 534 CHB patients with HCC who received curative hepatectomy were enrolled in this study (Figure 1). Liver cirrhosis was diagnosed by ultrasound findings as coarse liver parenchyma with nodularity, as well as small size of liver and the presence of features of portal hypertension [10]. HCC was defined according to the results of imaging studies and biochemical assays. Moreover, diagnosis was confirmed using histopathology. The diagnosis of HCC was based on the criteria of practice guidelines of the European Association for the Study of the Liver (EASL) or the American Association for the Study of Liver Disease (AASLD) [11, 12].

The baseline demographics, tumor status, serum biochemistries, and severity of liver diseases were comprehensively recorded at the time of diagnosis. HCC stage was defined according to the BCLC guidelines. The TNM classification was assessed according to the system of the International Union Against Cancer (7^th^ edition) [13]. Tumor differentiation was determined using the Edmondson grading system. After resection, patients were monitored regularly for serum *α*-fetoprotein (AFP) levels and were further assessed using abdomen ultrasonography or liver CT 3 to 6 months until death or dropout from the follow-up program. The follow-up was ended in December 2017. OS was defined as the interval between the dates of surgery and death or the interval between surgery and the last observation. The recurrence of HCC was diagnosed using liver CT or MRI.

### 2.2. Liver Fibrosis Evaluation

#### 2.2.1. Histology

After HCC resection, two experienced pathologists who were blinded to patient clinical information assessed liver specimens. The liver fibrosis stage was determined using the METAVIR fibrosis staging system, which was divided into five levels: F0—normal, F1—portal fibrosis, F2—fibrosis with few septa, F3—numerous septa, and F4—cirrhosis [14].

#### 2.2.2. FIB-4 Assessments

The FIB-4 values were calculated based on the laboratory parameters at the time of preoperative and postoperative 1-year liver resection as follows: age (years) × AST (U/L)/(platelets [10^9^/L] × (ALT [U/L]^1/2^)[15]. All patients were categorized based on the variation of the FIB-4 score (postoperative 1 year FIB‐4 − preoperative FIB‐4 score): the improved group (score ≤ 1), stable group (1 < score < 1), and deteriorated group (score ≥ 1).

#### 2.2.3. Statistical Analysis

Statistical analyses were performed using SPSS 21.0 (SPSS Company, Chicago, IL) for Windows. Experimental values of continuous variables were expressed as the means ± standard deviation. The chi-square test was used appropriately to evaluate the significance of differences between groups in data. The relationship between recurrence-free survival (RFS) and overall survival (OS) was analyzed using the Kaplan–Meier survival curves, and comparisons were determined using the log-rank test. Univariable and multivariable Cox proportional hazards regression models were used to estimate the effect of variables on the hazard of RFS and OS. Variables with *p* < 0.05 in the univariate analysis were incorporated into the multivariate analyses. The area under the receiver operating characteristic curve (AUROC) was used to estimate the predictive accuracy of the FIB-4 score. The FIB-4 score with the highest Youden's index (sensitivity + specificity − 1) yielded by the ROC analysis of diagnostic accuracies for OS was selected as the best cutoff value. A *p* value of <0.05 was considered statistically significant.

## 3. Result

### 3.1. Patient Characteristics

The characteristics of the population in this study before hepatectomy are presented in Table 1. A total of 534 patients who had resectable HCC from January 2001 to June 2016 were recruited in the current study: 453 (84.8%) males and 81 (15.2%) females. The mean age was 53.5 ± 11.2 years. The mean follow-up time was 65 months. A total of 249 patients (46.6%) had liver cirrhosis by ultrasound image, similar with pathologic diagnosis, in which 225 are F4 (46.2%, 225/487). In the Child-Pugh grade, the majority of patients were grade A (96%, 512/534). The mean FIB-4 score before operation was 2.3 ± 1.7.

### 3.2. FIB-4 Scores Are Correlated with Metavir Fibrosis Stage

**Figure 2:**
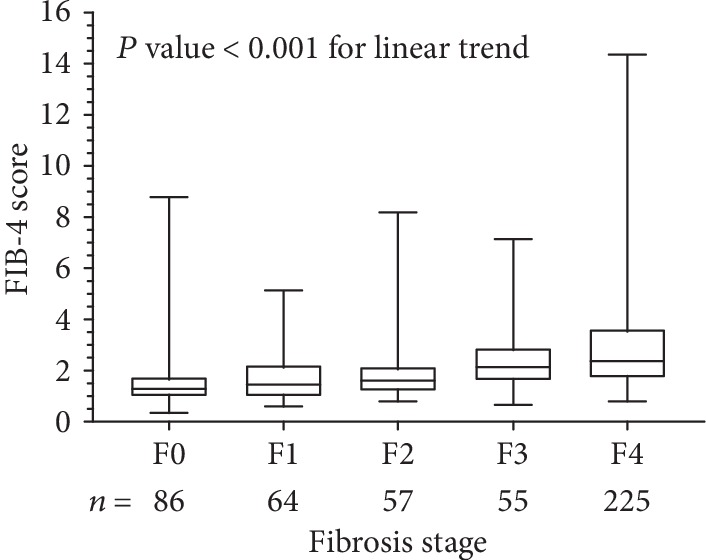
The association between FIB-4 score and fibrosis stage.

According to the METAVIR fibrosis stage, there were 86, 64, 57, 55, and 225 in F0, F1, F2, F3, and F4, respectively. The mean of the preoperative FIB-4 score was 1.6 ± 1.2, 1.8 ± 1.1, 1.9 ± 1.2, 2.3 ± 1.2, and 3.0 ± 2.0 in F0, F1, F2, F3, and F4 stages, respectively. The association between the preoperative FIB-4 level and Metavir fibrotic stage revealed a significant association (Spearman rho = 0.436, *p* < 0.01 for linear trend), resulting in higher median FIB-4 scores with increasing Metavir fibrosis stage (Figure 2).

### 3.3. ROC Curves of FIB-4 Score for HCC OS

The levels of the FIB-4 score were measured in 506 patients, and the median was 1.8 (mean 2.3, range 0.3–14.3, standard deviation 1.7). ROC curve analyses were performed to evaluate the predictive accuracy of FIB-4 for HCC OS, which indicated that a higher FIB-4 index could predict HCC OS with significantly more accuracy. The optimal cut-off point was 2, which corresponded to the maximum joint sensitivity and specificity on the ROC plot for FIB-4 (AUROC = 0.503, 95%CI = 0.521–0.634, *p* = 0.004). A sensitivity of 45% and a specificity of 73% were obtained for the prediction of death.

### 3.4. Pre- and Postoperative FIB-4 Score Is Associated with the Outcomes in Patients with Very Early and Early Stage HCC Received Resection

**Figure 3:**
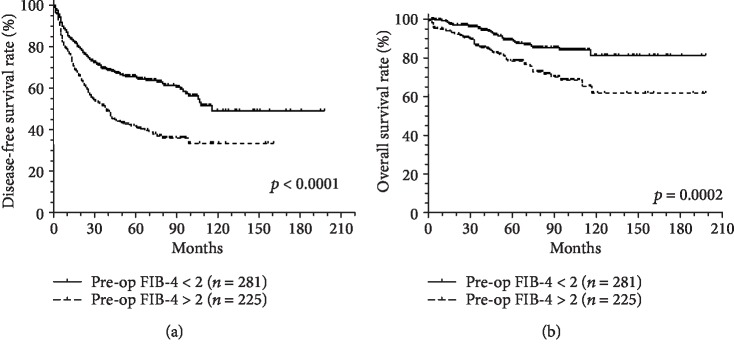
The preoperative FIB-4 score predicted the outcomes in patients with HCC after curative resection: (a) recurrence-free survival (RFS) and (b) overall survival (OS).

**Figure 4:**
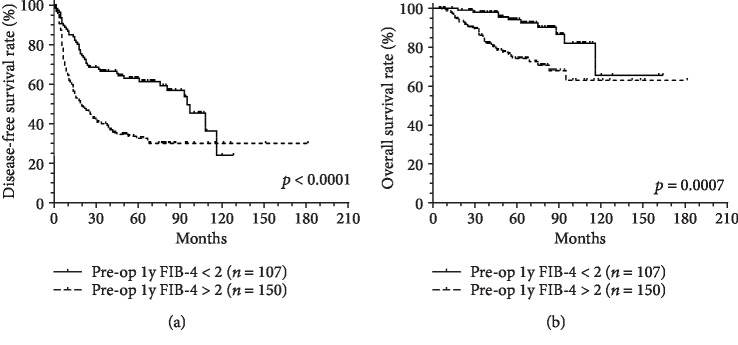
The postoperative FIB-4 score predicted the outcomes in patients with HCC after curative resection: (a) recurrence-free survival (RFS) and (b) overall survival (OS).

We investigated the predictive value of the pre- and postoperative FIB-4 score for all subjects. During the observation period (68 ± 41 months), 253 (47.4%) patients experienced recurrence, and 88 (16.5%) patients died. The 1-, 3-, and 5-year RFS rates were 81.2%, 62.8%, and 55.4%, respectively. The 1-, 3-, and 5-year OS rates were 97.2%, 92.1%, and 84.7%, respectively. Compared with the preoperative FIB-4 score≦2, patients with a preoperative FIB-4 score > 2 showed significantly worse RFS (*p* < 0.0001) and OS (*p* = 0.0002), respectively (Figure 3). We also compared the postoperative FIB-4 score, and the result was similar. Patients with a postoperative FIB-4 score > 2 had significantly worse RFS (*p* < 0.0001) and OS (*p* = 0.0007), respectively, compared with those with a postoperative FIB-4 score≦2 (Figure 4).

### 3.5. Prognostic Factors Associated with RFS

In RFS, univariate analysis identified the following factors as significantly linked to HCC recurrence: age > 60 years, presence of DM, presence of liver cirrhosis, platelet count < 150 × 10^9^/L, albumin < 3 g/dL, FIB − 4 > 2, tumor size > 5 cm, TNM stage, histology grade, and presence of vascular invasion (Table 2). Multivariate analysis revealed that DM (HR 1.887, 95% CI = 1.702–2.716, *p* = 0.001), liver cirrhosis (HR 2.117, 95% CI = 1.513–2.960, *p* < 0.001), FIB-4 > 2 (HR 1.085, 95% CI = 1.102–1.163, *p* = 0.022), tumor size > 5 cm (HR 1.615, 95% CI = 1.097–2.379, *p* = 0.015), histology stage (HR 3.306, 95% CI = 1.441–7.581, *p* = 0.005), and vascular invasion (HR 1.537, 95% CI = 1.132–2.088, *p* = 0.006) were independent risk factors for HCC recurrence. Subsequently, we analyzed the factors associated with RFS in patients without cirrhosis. Multivariate analyses showed that DM (HR 2.863, 95% CI = 1.584–5.194, *p* < 0.001), higher FIB-4 score (HR 1.219, 95% CI = 1.014–1.466, *p* = 0.035), and TNM stages (HR 3.786, 95% CI = 1.607–8.917, *p* = 0.002) were the independent risk factors (Table 3).

### 3.6. Prognostic Factors Associated with OS

In OS analysis, multivariate analysis disclosed that the presence of DM (HR 2.898, 95% CI = 1.628–5.160, *p* < 0.001), AFP > 20 ng/mL (HR 2.020, 95% CI = 1.164–3.507, *p* = 0.012), FIB-4 > 4 (HR 2.940, 95% CI = 1.622–5.329, *p* < 0.001), and histology stages (HR 6.400, 95% CI = 2.501–16.377, *p* < 0.001) were the independent risk factors (Table 4).

### 3.7. Change of the FIB-4 Score Predicts Outcomes of HCC Patients after Curative Resection

**Figure 5:**
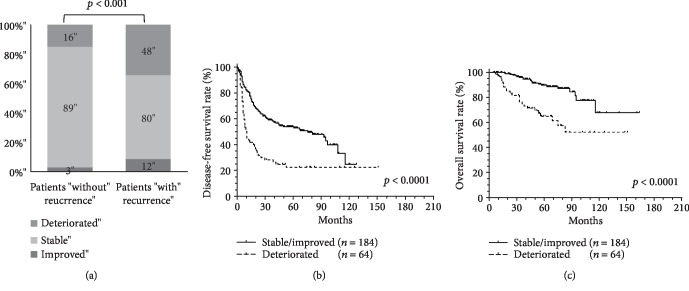
The change of the FIB-4 score predicted the outcomes in patients with HCC after curative resection. (a) The association between the change of the FIB-4 score and recurrence (b) in recurrence-free survival and (c) in overall survival.

The variations of the FIB-4 score between preoperation and postoperation at 1 year were classified into three groups: the improved group (*n* = 15), the stable group (*n* = 169), and the deteriorated group (*n* = 64). In patients without HCC recurrence, most patients (97.2%) had a stable or improved FIB-4 score, and only 2.8% of the patients had a deteriorated FIB-4 score; however, in patients with HCC recurrence, 8.6% of the patients had a deteriorated FIB-4 score (*p* < 0.01, Figure 5(a)). As shown in Figures 5(b) and 5(c), the patients with w stable or improved FIB-4 score had significant RFS and OS than those with a deteriorated FIB-4 score (*p* < 0.0001, both).

## 4. Discussion

HCC is one of the leading causes of cancer-related deaths worldwide [16], and in Asia, HBV infection is associated with most cases of cirrhosis and HCC [17], thus.

HBV-related HCC remains among the top causes of cancer mortality in Asian countries. Liver resection is a widely effective treatment for patients with resectable HCC and well liver function reserve; however, the long-term prognosis after resection of HCC remains unsatisfactory due to a high rate of intrahepatic recurrence [1, 18]. Hence, long-term monitoring of HCC progression after curative resection is important. So far, effective and reliable predictors for HCC prognosis after resection have not been identified. The serum *α*-fetoprotein (AFP) is the most common marker to follow up HCC after resection. However, less than half of the patients with HCC presented a raised AFP level. Except for AFP, the status of fibrosis or cirrhosis is also a useful predictor for HCC prognosis, which includes the Ishak stage [3] and liver stiffness measurement [19–21]. The FIB-4 score is used widely to evaluate the severity of cirrhosis in patients with HBV [7, 8], and a recent study reveals that the FIB-4 score is a good predictor for HBV-HCC patient after receiving operation [22]. But the postoperative FIB-4 score for HCC prognosis prediction is still unknown.

In our study, we calculated the preoperative and postoperative FIB-4 score of patients with HBV and had very early and early stage of HCC who had received curative HCC resection. A worse postoperative prognosis of OS and DFS is associated with a preoperative and postoperative FIB-4 score > 2 and a deterioration of the postoperative FIB-4 score by multivariate analysis. To explain this finding, comorbidity caused by the severity of liver cirrhosis and the condition of inflammation should be considered. Thrombocytopenia, which contributed to the elevation of the FIB-4 score, is a common comorbidity of liver cirrhosis and may be related to the occurrence of postoperative complication, such as bleeding or infection, and may contribute to a worse prognosis. Liver cirrhosis causes persistent inflammation of the liver tissue, and the production of inflammation cell may lead to a recurrence of HCC which is caused by circulating cancer cells [23]. Immune inflammation also plays a crucial role in advanced HCC by inducing the changes of the cancer microenvironment; thus, the use of an immune modulator may have potential benefit in patients who have HCC in the future [24]. Furthermore, a relapse HCC after operation may be due to the metastasis of *de novo* tumors, which arise in the cirrhotic liver rather than the original tumor [25]. In our study, we find that diabetes mellitus also contributed to a worse prognosis to OS and DFS. Similarly, a diabetes mellitus may represent a general inflammation condition of the patient and also lead to various comorbidities which may lead to a poor postoperative outcome. Thus, a good control of diabetes mellitus and inflammation may improve the patient's outcome. In a recent study by Shyu et al. [26], diabetes also led to a higher risk of hepatocellular carcinoma in patients with CHB. But the control of diabetes mellitus may not show a good prognosis to HCC instead; in a study by Casadei Gardini et al. [27] revealed a poor OS and progression-free survival in patients who had advanced HCC and under metformin treatment; resistance to cancer treatment caused by metformin was considered. Another study by Baba et al. [28] revealed insulin treatment promoted the progression of liver carcinogenesis in mice. Thus, while diabetes increased the risk of hepatocellular carcinoma and contributed to worsen the outcome in our study, the association between prognosis and a well control of diabetes needs further evaluation.

A recent study by Liao et al. [22] has a similar conclusion; in 108 HBV-HCC patients who received resection, the FIB-4 score can be a good predictor for outcome. A meta-analysis by Zhang et al. [29] also represented a result; in patients with HCC, the FIB-4 score may be a useful predictor. In our study, the value of the preoperative FIB-4 score is similar to the prior studies. In addition, we also found that the postoperative FIB-4 score and a deterioration of the postoperative FIB-4 score have a similar value to predict the HCC prognosis. To our knowledge, this is the first study indicating the value of the postoperative FIB-4 score. Therefore, we suggest that it is very important to monitor the FIB-4 score continuously for predicting the outcomes of HCC patients after resection.

The FIB-4 index, which is a simple and noninvasive liver fibrosis marker, was firstly developed for predicting the degree of liver fibrosis in patients with HCV/HIV coinfection [15], and subsequently, it was validated in a cohort of HBV-infected patients. Recent meta-analysis studies present that the FIB-4 index is helpful for predicting significant fibrosis in CHB patients [30, 31]. Our result is consistent with the prior studies. In the present study, we found that the FIB-4 score was associated with liver fibrosis stage, which was based on the histopathologic assessment after liver resection. Notably, in noncirrhosis patients (53.4%, 285/534), high levels of the FIB-4 index were significant predictions of HCC recurrence. These results support the notion that the FIB-4 index is a useful clinical marker to predict the outcomes of HCC patients with different fibrosis stages.

In the present study, except for the FIB-4 index, we also showed that DM, liver cirrhosis, tumor size, histology stage, and vascular invasion were important predictors for HCC recurrence; in addition, DM, AFP, and histology stage were independent risk factors for overall survival. It was consistent with the results from the previous studies in which patient factors (DM) [32], liver background factor (liver cirrhosis) [33], and tumor factor (vascular invasion, histology stage, and AFP) [34] determined the outcomes of HCC patients.

Regarding HBV antiviral therapy, several studies have showed that nucleoside analogue treatment was associated with a lower risk factor of HCC recurrence after resection [35, 36]. But there are still some studies indicating the opposite results [37]. In the present study, there was no association among antiviral therapy, RFS, and survival. The result is not very solid due to the variations and complex of antiviral therapies in our cohort. In Taiwan, the HBV treatment is covered by the National Health Insurance, but HCC is not indicated, unless patients were cirrhotic with serum HBV DNA > 2000 IU/mL. Therefore, many patients were treated by self-paid antiviral agents, which resulted in the poor compliance. Hence, it is too weak to make a conclusion here that antiviral therapy is not associated with RFS and OS. However, we still can see the better RFS and OS trend in the later era (2009-2016) compared with the early era (2001-2008), although there are no significant changes in both, which suggested that the nucleoside analogue treatment changed this. This result is compatible with the study from Taiwan which used the Taiwan National Health Insurance Research Database enrolling 4569 HBV-related HCC patients [35].

There are some limitations in our study. First, we only enrolled patients with HBV; whether our results could be applied to CHC patients needs further research. Second, we retrospectively collected the data from medical records. Some patients lost to follow up or even died after operation. We should interpret the data in our study with caution. Third, our patient population is mainly Asians. All of the patients received operation in a single medical center. Thus, the conclusion in our study may not fit other race or patients who received operation in other regions. Finally, all patients in this cohort were treated at a tertiary medical center; therefore, referral bias could not be completely avoided. Hence, future well-designed and larger studies will be needed to consolidate our results.

In conclusion, the pre- and postoperative FIB-4 indexes are useful clinical markers to predict the outcomes in HBV-HCC patients after curative hepatectomy. Therefore, the FIB-4 index should be assessed regularly for HCC patients before and after resection. Further studies are warranted before it can be applied into the daily practice.

## Figures and Tables

**Table 1:** Baseline characteristics of the study population (*n* = 534).

Age (years; mean ± SD)	53.5 ± 11.2
Male, n (%)	453 (84.8%)
Bilirubin (g/dL; mean ± SD)	0.9 ± 0.3
Albumin (g/dL; mean ± SD)	3.8 ± 0.6
AFP (>15 ng/mL), *n* (%)	261 (50.3%)
Liver cirrhosis, *n* (%)	249 (46.6%)
Metavir fibrosis (F0 : F1 : F2 : F3 : F4)	86 : 64 : 57 : 55 : 225
Tumor size (>5 cm), *n* (%)	96 (18.0%)
Tumor number (single : multiple)	507 : 27
TNM stage (I : II : III)	285 : 208 : 41
Child-Pugh grade (A : B)	512 : 22
MELD score (mean ± SD)	8.0 ± 3.1
FIB-4 score	2.3 ± 1.7
*Pathological features*	
Micro-/macrovascular invasion, *n* (%)	221 (41.4%)
Histological grade (I : II : III)	70 : 447 : 17

MELD = model for end-stage liver disease; FIB-4 = fibrosis-4.

**Table 2:** Univariate and multivariate analysis of prognostic factors for RFS in CHB-HCC patients after curative hepatectomy.

Variable	Comparison	Univariate		Multivariate	
		HR (95% CI)	*p* value	HR (95% CI)	*p* value
Age (years)	>60 vs. ≦60	1.345 (1.042–1.736)	0.023		
Sex	Male vs. female	0.970 (0.689–1.364)	0.860		
DM	Yes vs. no	2.036 (1.493–2.778)	<0.001	1.887 (1.702–2.716)	0.001
AFP (ng/mL)	>20 vs. ≦20	1.214 (0.945–1.559)	0.130		
Platelet (10^9^/L)	≦150 vs. >150	1.408 (1.091–1.816)	0.009		
Albumin (g/dL)	≦3 vs. >3	1.617 (1.076–2.429)	0.021		
Liver cirrhosis	Yes vs. no	2.073 (1.612–2.668)	<0.001	2.117 (1.513–2.960)	<0.001
MELD score	>14 vs. ≦14	0.967 (0.430–2.174)	0.935		
Child-Pugh grade	B vs. A	1.400 (0.764–2.563)	0.276		
FIB-4 > 2	>2 vs. ≦2	1.902 (1.491–2.460)	<0.001	1.085 (1.102–1.163)	0.022
Tumor size (cm)	>5 vs. ≦5	1.370 (1.017–1.849)	0.039	1.615 (1.097–2.379)	0.015
Tumor no.	Multiple vs. single	1.395 (0.840–2.316)	0.198		
TNM stages	III vs. I + II	1.599 (1.072–2.385)	0.021		
Histology stages	III vs. I+II	1.893 (1.005–3.565)	0.048	3.306 (1.441–7.581)	0.005
Vascular invasion	Yes vs. no	1.536 (1.197–1.970)	0.001	1.537 (1.132–2.088)	0.006

DM = diabetes mellitus; MELD = model for end-stage liver disease; FIB-4 = fibrosis-4.

**Table 3:** FIB-4 score predicted the recurrence in noncirrhotic CHB-HCC patients after resection.

Variable	Comparison	Multivariate	
		HR (95% CI)	*p* value
DM	Yes vs. no	2.863 (1.584–5.194)	<0.001
FIB-4 score	Increase 1	1.219 (1.014–1.466)	0.035
TNM stages	III vs. I+II	3.786 (1.607–8.917)	0.002

**Table 4:** Univariate and multivariate analysis of prognostics factors for OS in CHB-HCC patients after curative hepatectomy.

Variable	Comparison	Univariate		Multivariate	
		HR (95% CI)	*p* value	HR (95% CI)	*p* value
Age (years)	>60 vs. ≦60	1.411 (0.949–2.167)	0.116		
Sex	Male vs. female	1.096 (0.596–2.017)	0.767		
DM	Yes vs. no	3.021 (1.889–4.832)	<0.001	2.898 (1.628–5.160)	<0.001
AFP (ng/mL)	>20 vs. ≦20	1.609 (1.037–2.498)	0.034	2.020 (1.164–3.507)	0.012
Platelet (10^9^/L)	≦150 vs. >150	1.539 (1.002–2.363)	0.049		
Albumin (g/dL)	≦3 vs. >3	1.920 (1.018–3.622)	0.044		
Liver cirrhosis	Yes vs. no	1.937 (1.259–2.980)	0.003		
MELD score	>14 vs. ≦14	1.443 (0.455–4.574)	0.534		
Child-Pugh grade	B vs. A	2.328 (1.017–5.374)	0.045		
FIB-4 > 2	>2 vs. ≦2	2.207 (1.420–3.429)	<0.001	2.940 (1.622–5.329)	<0.001
Tumor size (cm)	>5 vs. ≦5	1.385 (0.846–2.268)	0.195		
Tumor no.	Multiple vs. single	0.926 (0.358–2665)	0.963		
TNM stages	III vs. I+II	3.069 (1.804–5.223)	<0.001		
Histology stages	III vs. I+II	6.069 (3.129–11.779)	<0.001	6.400 (2.501–16.377)	<0.001
Vascular invasion	Yes vs. no	1.906 (1.248–2.911)	0.003		

DM = diabetes mellitus; MELD = model for end-stage liver disease; FIB-4 = fibrosis-4.

## Data Availability

The excel data used to support the findings of this study are currently under embargo while the research findings are commercialized. Requests for data, 12 months after the publication of this article, will be considered by the corresponding author.

## Abbreviations

FIB-4: Fibrosis-4
CHB: Chronic hepatitis B
HBV: Hepatitis B virus
HCC: Hepatocellular carcinoma.
